# Immunomodulatory properties of mesenchymal stem cells/dental stem cells and their therapeutic applications

**DOI:** 10.1038/s41423-023-00998-y

**Published:** 2023-03-27

**Authors:** Peishan Li, Qianmin Ou, Songtao Shi, Changshun Shao

**Affiliations:** 1 The First Affiliated Hospital of Soochow University, State Key Laboratory of Radiation Medicine and Protection, Institutes for Translational Medicine, Soochow University, Suzhou, PR China; 2 South China Center of Craniofacial Stem Cell Research, Hospital of Stomatology, Guangdong Provincial Key Laboratory of Stomatology, Sun Yat-Sen University, Guangzhou, PR China

**Keywords:** Mesenchymal stem cells, Dental stem cells, Immunoregulation, Inflammation, Inflammatory diseases, Gene regulation in immune cells

## Abstract

Mesenchymal stem/stromal cells (MSCs) are widely distributed in the body and play essential roles in tissue regeneration and homeostasis. MSCs can be isolated from discarded tissues, expanded in vitro and used as therapeutics for autoimmune diseases and other chronic disorders. MSCs promote tissue regeneration and homeostasis by primarily acting on immune cells. At least six different types of MSCs have been isolated from postnatal dental tissues and have remarkable immunomodulatory properties. Dental stem cells (DSCs) have been demonstrated to have therapeutic effects on several systemic inflammatory diseases. Conversely, MSCs derived from nondental tissues such as the umbilical cord exhibit great benefits in the management of periodontitis in preclinical studies. Here, we discuss the main therapeutic uses of MSCs/DSCs, their mechanisms, extrinsic inflammatory cues and the intrinsic metabolic circuitries that govern the immunomodulatory functions of MSCs/DSCs. Increased understanding of the mechanisms underpinning the immunomodulatory functions of MSCs/DSCs is expected to aid in the development of more potent and precise MSC/DSC-based therapeutics.

## Introduction

In almost every vertebrate organ, there are mesenchymal stromal cells (MSCs). Because these cells possess the capacities of self-renewal and are progenitors that can give rise to adipocytes, chondrocytes, osteoblasts and myofibroblasts in response to differentiation or inflammatory cues, they are frequently referred to as mesenchymal stem cells (also abbreviated MSCs). Due to their multilineage differentiation potential, ability to regulate the immune response, easy accessibility to source tissues and excellent propagation capacity in vitro, MSCs have been regarded as an ideal source for therapeutics in tissue regeneration and autoimmune and hyperinflammatory diseases. MSCs are a highly heterogeneous cell type with multiple postnatal tissue origins. These cells originate either from the mesoderm or ectoderm [1–4]. The distinct differences in their lineage, development and tissue distribution can not only determine their functional diversity but also have implications in clinical applications. Because MSCs derived from nondental tissues such as bone marrow, adipose tissues and umbilical cord have been extensively reviewed elsewhere [5–8], this review will focus on MSCs derived from dental tissues, although some general mechanistic aspects of MSC biology will be presented.

**Fig. 1:**
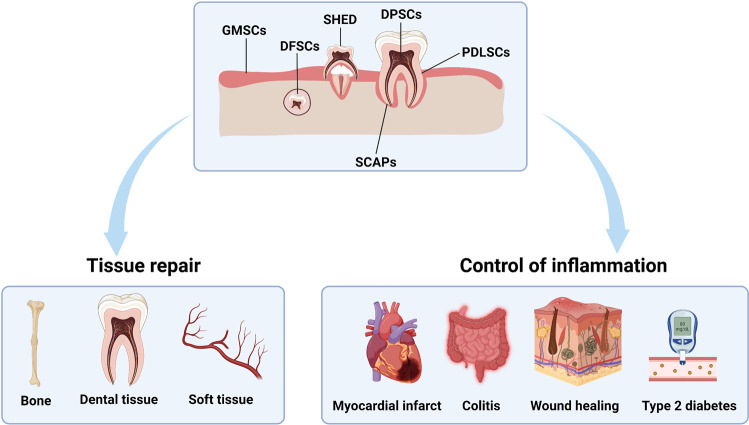
The classification and therapeutic applications of DSCs. Six different types of DSCs have been isolated and characterized, including stem cells from dental pulp (DPSCs), periodontal ligament (PDLSCs), deciduous teeth (SHED), apical papilla (SCAPs), dental follicles (DFSCs), and gingiva (GMSCs). DSCs have therapeutic potential for tissue repair (bone regeneration, dental tissue regeneration and soft tissue reconstruction) and inflammatory diseases (myocardial infarct, colitis, wound healing and type 2 diabetes)

To date, at least six different types of postnatal dental tissue-derived MSCs have been isolated and characterized, including stem cells from dental pulp (DPSCs), periodontal ligament (PDLSCs), deciduous teeth (SHED), apical papilla (SCAPs), dental follicles (DFSCs), and gingiva (GMSCs) (Fig. 1). STRO-1 and CD146 may serve as reliable surface markers for dental stem cells (DSCs). Due to their easy access, excellent propagation capacity in vitro and potent immunoregulatory properties, DSCs have been used in some preclinical studies and clinical trials for treating hyperinflammatory disorders, neurodegenerative diseases, organ injuries, autoimmune diseases, orthopedic disorders and diabetes (Table 1). Conversely, MSCs derived from nondental tissues such as umbilical cord and adipose tissue are being explored for their therapeutic benefits in the treatment of periodontitis and other dental diseases. This review summarizes the main therapeutic uses of MSCs/DSCs, highlights their mechanisms of action in immunoregulation, and discusses the extrinsic inflammatory cues that elicit the immunoregulatory properties and the intrinsic metabolic circuitries in these cells. We also provide some perspectives on possible strategies that can augment the therapeutic effects of MSCs/DSCs.

**Table 1:** Clinical trials for DSCs (source: https://www.clinicaltrials.gov/)

Disease	Infusion method	Sample size	Cell mass	Cell source	Study phase	NCT number	Location
COVID-19	Intravenously infusion	20	3 × 10^7 cells	DPSCs	Recruiting	NCT04336254	China
Periodontitis	Local injection	36	1 × 10^6–4 × 10^7 cells	DPSCs	Recruiting	NCT04983225	China
Acute Ischemic Stroke	Intravenously infusion	79	1 or 3 × 10^8 cells	DPSCs	Completed	NCT04608838	Japan
Cleft Lip and Palate	Local implantation	62	N/A	DPSCs	Completed	NCT03766217	Brazil
Knee Osteoarthritis	Intraarticular injection	60	N/A	DPSCs	Unknown	NCT04130100	China
Periodontal Diseases	Local implantation	29	N/A	DPSCs	Completed	NCT03386877	Italy
Periodontal Diseases	Local injection	40	1 × 10^6 cells	DPSCs	Unknown	NCT02523651	China
Periodontal tissue regeneration	Local implantation	30	N/A	PDLSCs	Completed	NCT01357785	China
Periodontitis	Local implantation	80	N/A	PDLSCs	Completed	NCT01082822	China
Periodontal Intrabony Defect	Local implantation	20	N/A	GMSCs	Completed	NCT03638154	Egypt
Periodontitis	Local implantation	30	N/A	GMSCs	Unknown	NCT03137979	China
Liver Cirrhosis	Peripheral vein infusion	40	1 × 10^6 cells/kg	SHEDs	Unknown	NCT03957655	China
Cleft Lip and Palate	Local implantation	5	N/A	SHEDs	Completed	NCT01932164	Brazil
Type 2 Diabetes	Intravenous drip	24	0.1 IU/kg	SHEDs	Completed	NCT03658655	China
Dental Pulp Necrosis	Local implantation	80	N/A	SHEDs	Unknown	NCT01814436	China
Type 1 Diabetes	Intravenously infusion	24	0.11 IU/kg	SHEDs	Unknown	NCT03912480	China

### Ontogeny and differentiation of DSCs

MSCs residing in most internal organs are primarily derived from the mesoderm. DSCs originate from the neural crest. Despite their distinct origins, MSCs/DSCs all possess general properties such as self-renewal, multipotent differentiation, immunomodulation, and the expression of a set of MSC surface markers [9]. However, DSCs tend to have stronger proliferative and neural differentiation potential [10], which is beneficial in the regeneration of neuronal tissue and the treatment of neurodegenerative diseases, such as Parkinson’s disease and Alzheimer’s disease [11]. Feng et al. showed that NG2^+^ pericyte-derived DSC precursors contributed to odontoblast differentiation during dental pulp development and tissue regeneration [12, 13]. Genetic lineage-tracing studies showed that DPSCs originated from peripheral nerve-associated glia, and the glia-to-mesenchymal cell transition pattern may be unique to the lineage evolution of DPSCs [14]. Zhao et al. reported that Gli1^+^ MSCs surrounding the cervical loop of the incisor are the progenitor cells of PDLSCs and DPSCs, and Gli1^+^ cells within the suture mesenchyme are the major MSCs responsible for craniofacial bone development and homeostasis [15, 16]. CD24a^+^ SCAPs are multipotent tooth root stem cells that can regenerate tooth roots with functional dentin and neurovascular-like structures [17]. These studies show that DSCs have multiple subsets.

DSCs exhibit increased odontogenic preference but have reduced chondrogenic and adipogenic differentiation potential [18]. The differentiation of DSCs is regulated by multiple signaling pathways, such as the BMP/TGF-β and WNT/β-catenin pathways. BMP/TGF-β signaling activity is required for the odontogenic differentiation of DSCs and root development [19]. Specifically, the loss of BMP/TGF-β signaling in DSCs leads to a delay in tooth eruption, defects in root elongation, and failure of extracellular matrix formation [20]. During tooth development, DSCs give rise to transit amplifying cells, which then differentiate into different cell types, including odontoblasts, cementoblasts and dental pulp cells [21]. The WNT/β-catenin pathway plays an important role in the maintenance of DSCs by transit amplifying cells (TACs), and the loss of Wnt5a results in the loss and impaired differentiation of DSCs and diminished TACs [21].

DSCs can be isolated from different parts of the tooth, and accumulating evidence has shown functional differences among DSCs in terms of proliferation rate, osteo/odonto differentiation rate and angiogenic potential [22]. Therefore, tissue-specific markers are needed, and the functions of the different types of DSCs need to be further explored. The advancement of single-cell lineage tracing technology holds great promise in elucidating the heterogeneity and specific functions among different types of DSCs.

### Interactions of DSCs with the tissue microenvironment

Previous studies have shown that the regenerative and immunomodulatory activity of DSCs is dynamically regulated by the stem cell niche and immune microenvironment [23, 24]. PDLSCs isolated from inflamed tissue showed increased proliferation rates but decreased osteogenic potential [25, 26]. Further study suggested that periodontitis could undermine the osteogenic ability of PDLSCs via the UCHL1/BMP2/Smad signaling pathway, accelerating bone resorption [27]. PDLSCs can undergo gasdermin-D (GSDMD)-dependent pyroptosis, leading to periodontitis by increasing IL-1β release, enhancing inflammation, and promoting osteoclastogenesis [28]. These studies suggest that the properties of DSCs vary with their microenvironment.

DSCs exert immunosuppressive effects mostly through the secretion of immunoregulatory cytokines, which modulate innate and adaptive immunity, as well as the complementary system [29]. Thus, the reciprocal interactions between DSCs and immune cells can help maintain tissue homeostasis and avoid excessive inflammatory responses. When tissue injury occurs, DSCs can react rapidly via their immunomodulatory activities and prevent the exacerbation of injury [24, 30]. DPSCs from inflammatory pulpitis (I-DPSCs) were shown to suppress the proinflammatory functions of macrophages through the TNF-α/IDO axis, attenuating the exacerbation of pulpitis [31]. Compared to DPSCs from healthy pulp tissue, I-DPSCs expressed increased levels of IL-6. I-DPSCs and DPSCs treated with IL-6 have impaired neurogenic potential [32]. I-DPSCs were also shown to have diminished immunosuppressive properties [33]. The periodontitis microenvironment compromises the immunomodulatory effects of PDLSCs, leading to the accumulation of inflammatory immune cells in periodontal bone [25]. GMSC-derived exosomes in the inflammatory microenvironment can enhance M2 macrophage polarization and inhibit periodontal bone loss [34]. Therefore, these studies indicate that the regenerative and immunomodulatory activities of DSCs are vital for dental tissue homeostasis.

### Current therapeutic applications of harvested MSCs/DSCs in tissue repair and the control of inflammation

#### Tissue repair

Compared with mesoderm-derived MSCs, neural crest-derived MSCs show increased differentiation potential to neural cells and chondrocytes, as well as increased immunomodulatory and anti-inflammatory effects in vitro and in vivo, suggesting that DSCs have great therapeutic potential for applications in tissue repair, including bone, dental and soft tissues [35].

A previous study demonstrated that PDLSCs showed a stronger tissue regeneration capacity than MSCs derived from bone marrow (BM-MSCs) on critical-size defects in the immunodeficient rat calvarium, suggesting the superiority of using PDLSCs in bone tissue regenerative therapy [36]. The transplantation of SHED can ameliorate secondary osteoporosis and promote bone regeneration in mice with systemic lupus erythematosus [37]. DPSC-derived extracellular matrix enhances artificial bone integration and promotes artificial bone regeneration and repair; thus, DPSC-derived extracellular matrix can be used to decorate various biomaterials in bone tissue engineering [38]. Another study reported that implanted DPSCs could survive in the defect area and accelerate the regeneration of calvarial bone *via* an endochondral bone ossification-like process [39]. These studies showed that DSCs may promote bone tissue repair through multiple mechanisms.

In dental tissue repair, DSCs can generate dental-related tissue after transplantation in vivo. Gronthos et al. reported that DPSCs could regenerate a dentin-pulp complex, which is composed of mineralized matrix and fibrous tissue [40]. Functional cementum/PDL-like structures can be regenerated after PDLSCs are transplanted into immunocompromised mice, which is vital for alveolar bone regeneration and periodontitis treatment [10]. In general, DSCs have potential in bone tissue regeneration and repair, especially in the oral and maxillofacial areas.

In addition to their applications in bone regeneration, DSCs are also recommended for soft tissue reconstruction, such as in periodontal ligament, dental pulp and nerve tissue [41–43]. Because of their origin from the neural crest and their residence in a neurovascular niche, DSCs have the potential to re-establish neurovascular inductive activity [44]. Extracellular vesicles (EVs) derived from GMSCs were reported to promote the regeneration of periodontal ligament in damaged periodontal tissue [45]. SHEDs and DPSCs possess strong neurogenic and angiogenic abilities and are thus optimal candidates for dental pulp regeneration [46]. SHEDs were administered to minipigs and regenerated physiologic pulp patterns with odontoblasts lining the dentin wall using cell aggregate technology. Similarly, DPSC aggregates form pulp-like tissues with rich blood vessels within the human root canal 6 weeks after implantation [47]. Importantly, DPSCs may serve as a better choice for treating Parkinson’s disease than BM-MSCs due to their predisposition toward neural differentiation and their potential to regenerate neurons [48]. A recent study showed that Nestin^+^ DPSCs could survive for 1 month after transplantation in the brains of nude mice and form complete blood vessels that integrated into the host cerebrovascular system [49].

#### Control of inflammation

The immunomodulatory property of DSCs makes them suitable therapeutics for aberrant immune and inflammatory diseases. DSCs can exert their immunomodulatory effects through direct and indirect mechanisms [50, 51]. DPSCs induce apoptosis in activated T cells in vitro and reduce inflammatory tissue damage in mice with colitis, which is related to the expression of Fas ligand (FasL) by DPSCs. Downregulation of FasL expression in DPSCs compromises their immunomodulatory properties [52]. Conditioned medium (CM) from DPSCs effectively improved healing and attenuated the inflammatory response of skin fibroblasts [53]. Systemic infusion of GMSCs significantly ameliorated colonic inflammation, restored injured gastrointestinal mucosal tissues, reversed diarrhea and weight loss, and suppressed overall disease activity in mice with experimental colitis [54]. GMSCs are capable of polarizing macrophages into the M2 phenotype, which is characterized by upregulated expression of CD206, increased secretion of the anti-inflammatory cytokine IL-10 and phagocytotic activity [55]. Unlike murine BM-MSCs, which are impaired in their migration and anti-inflammatory response with aging [56], human GMSCs have been shown to retain stable karyotypes and immunomodulatory characteristics independent of donor age [57, 58]. DFSCs can also reduce inflammation through the paracrine factors TGF-β3 and TSP-1 [59].

The administration of SHED-derived CM reduces myocardial infarct size and inflammatory cytokine levels, such as TNF-α, IL-6, and IL-β [60]. The anti-inflammatory effect of SHED-CM is superior to that of CM derived from BM-MSCs and adipose-derived stem cells (ADSCs) [60]. DPSC-CM alleviates Sjögren’s syndrome by promoting Treg cell differentiation and inhibiting Th17 cell differentiation in the mouse spleen [61]. DPSC-EVs can reduce the levels of inflammatory cytokines and senescence-associated secretory phenotypic factors, thus reversing oxidative stress in submandibular cells and preventing irradiation-induced cellular senescence [62]. DPSC-EVs can improve ischemia‒reperfusion in mice through anti-inflammatory mechanisms mediated by the HMGB1/TLR4/MyD88/NF-κB pathway [63]. Therefore, DSCs have outstanding anti-inflammatory functions that can be harnessed for therapeutic applications in inflammatory diseases.

#### Clinical Studies

Since the safety and efficacy of DSCs have been extensively verified, DSCs are increasingly being used in clinical studies. Currently, there are more than 15 clinical trials for tissue regeneration and disease repair using DSCs (Table 1), including DPSCs for COVID-19 and SHEDs for dental pulp regeneration. Some clinical studies have yielded exciting outcomes. Chen et al. conducted a randomized trial using autologous PDLSCs for periodontal intrabony defects and validated the safety of PDLSCs in clinical applications, which is the first study of DSCs in a clinical trial [64]. Xuan et al. implanted ex vivo-expanded autologous SHEDs in patients with pulp necrosis [65]. The implanted SHEDs regenerated three-dimensional whole dental pulp with an odontoblast layer, blood vessels, and nerves, which represents the first human organ that was successfully regenerated by DSCs in randomized clinical trials [65]. Li et al. reported that SHED infusion was effective in improving glucose metabolism and islet function in type 2 diabetes mellitus patients, which is the first successful study using DSCs to treat systemic disease [66]. Ferrarotti et al. reported that the delivery of DPSCs into intrabony defects in a collagen scaffold significantly improved the clinical parameters of periodontal regeneration 1 year after treatment [67]. Thus, the clinical benefits of DSCs may be attributed to their regenerative capacity, as well as their ability to restore tissue immune homeostasis.

### The immunoregulatory pathways of MSCs/DSCs

**Fig. 2:**
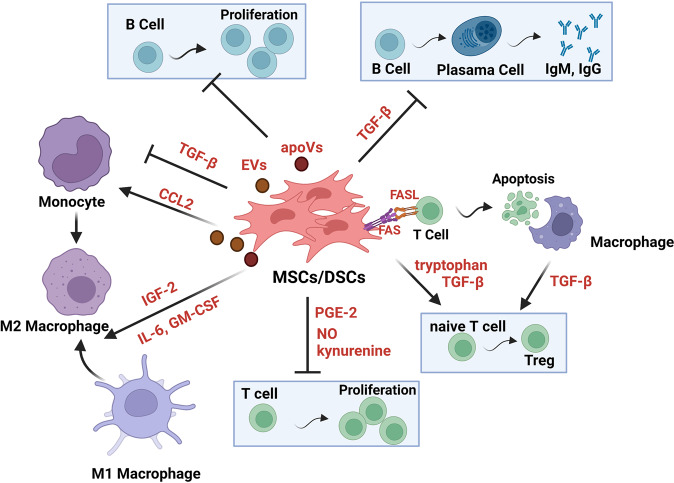
The main routes through which MSCs/DSCs exert their immunoregulatory effects. MSCs/DSCs exert their immunomodulatory effects by producing metabolites, cytokines, growth factors, chemokines, extracellular vesicles, and apoptotic vesicles and T-cell death-mediated immunoregulation. MSCs/DSCs can suppress T-cell and B-cell proliferation, promote naive CD4^+^ T cells to differentiate into Treg cells, instruct macrophages to acquire an immunosuppressive phenotype, or inhibit the production of IgM and IgG. In addition, MSCs/DSCs induce T-cell apoptosis and subsequently trigger macrophages to produce TGFβ, which induces the differentiation of Tregs and immune tolerance

A few studies have demonstrated that in vitro expanded MSCs/DSCs can facilitate the repair and regeneration of damaged tissue through their immunomodulatory actions [7]. MSCs/DSCs possess strong immunomodulatory abilities, which endows MSCs/DSCs with therapeutic potency in various degenerative and inflammatory disorders [68]. The immunomodulatory effects of MSCs/DSCs are exerted via the production of metabolites, cytokines, growth factors, chemokines, EVs, and apoptotic vesicles and T-cell death-mediated immunoregulation (Fig. 2). Below, we highlight some of the main pathways that contribute to the immunomodulation mediated by MSCs/DSCs.

#### Metabolites: NO, tryptophan metabolites, PGE2

It has been demonstrated that when exposed to an inflammatory environment, MSCs can orchestrate local and systemic innate and adaptive immune responses. Inflammation-primed MSCs express inducible nitric oxide synthase (iNOS) in rodents (such as mice, rats, hamsters and rabbits) or indoelmine-2–3-dixoygenase (IDO) in other mammalian species to suppress T-cell responsiveness [7, 69]. Murine MSCs express high levels of iNOS in response to activation by proinflammatory cytokines and produce NO, which is one of the major mediators of T-cell suppression by MSCs. MSCs from inducible NOS^−/−^ mice had reduced abilities to suppress T-cell proliferation [70]. Moreover, IL-17 dramatically enhanced the immunosuppressive effects of MSCs by promoting iNOS expression in MSCs. This reinforcing effect of IL-17 was mediated by enhancing mRNA stability by downregulating ARE/poly(U)-binding/degradation factor 1 (AUF1), a well-known factor that promotes mRNA decay [71]. However, NO-mediated immunosuppression by MSCs may become immune-enhancing under inadequate stimulation. When iNOS production was inhibited or genetically ablated, MSCs strongly enhanced T-cell proliferation in vitro and the delayed-type hypersensitivity response in vivo. It is likely that in the absence of NO, MSC-produced chemokines can still attract immune cells [72].

The immunosuppressive effects of human MSCs in response to stimulation with IFN-γ plus TNF-α or IL-1 primarily rely on IDO. IDO is a catabolic enzyme that degrades tryptophan to kynurenine. Depletion of local tryptophan by IDO activates the stress-response kinase general control nonderepressible 2 (GCN2), which senses amino acid withdrawal. GCN2 activation inhibits T-cell proliferation and biases naive CD4^+^ T cells toward differentiation into Treg cells [73, 74]. Moreover, the soluble factors (kynurenine and downstream metabolites) generated by IDO can bind and activate the aryl hydrocarbon receptor, which can also promote Treg cell differentiation [75] and induce dendritic cells to adopt an immunosuppressive phenotype [76].

A recent study showed that local injection of ADSCs significantly reduced alveolar bone loss and the ratio of iNOS^+^/CD206^+^ macrophages in a rat model of periodontitis [77]. IDO expression in ADSCs was upregulated by inflammatory stimuli and was required for the therapeutic effects of ADSCs in experimental periodontitis. Mechanistically, ADSCs release kynurenine, which is a tryptophan metabolite catalyzed by IDO, to activate the aryl hydrocarbon receptor and enhance its downstream target *NFE2L2* in macrophages. *NFE2L2-*encoded NRF2 not only functions as a master regulator of antioxidant defense but also represses the expression of inflammatory genes. As expected, NRF2 upregulation in macrophages was inhibited by inhibiting IDO and 1-methyltryptophan (1-MT), and the anti-inflammatory effect of ADSCs on macrophages was blocked when NRF2 expression in macrophages was silenced. Kynurenic acid, another IDO-derived metabolite that shares the same aryl hydrocarbon receptor as kynurenine, can promote TNF-α-stimulated gene-6 (TSG-6) expression and alleviate neutrophil infiltration in injured lungs [78].

In addition to iNOS and IDO, the immunosuppressive properties of MSCs are also mediated through the expression of COX1/COX2 enzymes and the production of prostaglandin E2 (PGE2). While COX1 is constitutively expressed in most mammalian cells, COX2 is an inducible enzyme and cab ne induced by proinflammatory cytokines and growth factors [79]. PGE2 has been reported to mediate the immunoregulatory function of MSCs via the induction of IL-10 secretion by macrophages [80], suppression of NK cell cytotoxic function [81] and suppression of Th17 differentiation [82].

DSCs exert their regulatory effects on inflammation similar to MSCs derived from other tissues. GMSCs specifically suppress peripheral blood lymphocyte proliferation and induce the expression of a wide range of immunosuppressive factors, including IDO, iNOS, and COX-2, in response to inflammatory cytokines [54]. PGE2 plays a crucial role in PDLSC-mediated immunomodulation and periodontal tissue regeneration in a miniature pig periodontitis model [83]. GMSC-induced blockade of de novo synthesis of proinflammatory cytokines by mast cells is also mediated by PGE2 [84].

#### Growth factors, cytokines and chemokines

MSCs secrete a variety of growth factors, which not only function as effector molecules during tissue repair but also regulate the differentiation of MSCs themselves. During adipose tissue injury and repair, fibroblast growth factor-2 strongly promotes ADSC proliferation and HGF secretion through the c-Jun N-terminal kinase signaling pathway. HGF expression in MSCs contributes to the regeneration of adipose tissue and suppression of fibrogenesis after injury [85]. The secretion of VEGF-C by MSCs was greatly increased by pretreatment with the inflammatory cytokines IFN-γ and TNF-α and played a key role in mediating the wound healing effect of MSCs [86]. In addition, VEGF-C treatment significantly increased the expression of RUNX2 and osteogenic marker genes and the mineralization of MSCs [87]. DPSCs overexpressing hepatocyte growth factor were shown to facilitate the repair of DSS-induced ulcerative colitis [88].

MSCs can produce insulin-like growth factor 2 (IGF-2) to preprogram macrophages to undergo anti-inflammatory polarization during their maturation [89]. When exposed to low concentrations of IGF-2, maturing macrophages are committed to oxidative phosphorylation (OXPHOS) and consequently increase the expression of PD-L1, which is required for the beneficial effect of IGF-2 on experimental autoimmune encephalomyelitis. Further study showed that IGF-2 at low concentrations conferred anti-inflammatory properties to maturing macrophages by binding to IGF2R, which resulted in its nuclear translocation, the activation of GSK3 α/β, and Dnmt3a-mediated repression of v-ATPase. Due to the lack of v-ATPase, protons are rerouted to mitochondria from lysosomes, enabling the increase in OXPHOS that is characteristic of anti-inflammatory macrophages [90].

Similar to BM-MSCs, which are capable of reprogramming macrophages into the M2 phenotype [91], GMSCs can also promote the polarization of PBMC-derived macrophages toward the M2 phenotype. IL-6 and GM-CSF synergistically contribute to the induction of M2 macrophages in coculture with GMSCs [92]. A comparison of DPSCs and DFSCs isolated from the same tooth showed that DFSCs proliferated faster than DPSCs, while the latter produced more transforming growth factor (TGF)-β and suppressed the proliferation of peripheral blood mononuclear cells [93]. DPSCs were also shown to inhibit acute allogeneic immune responses by releasing TGF-β, which inhibits the production of IgM and IgG by allogeneic activation of responder B lymphocytes [94]. Moreover, similar to DPSCs, DPSCs isolated from inflamed pulp can suppress macrophage functions via the TNF-α/IDO axis, thereby providing a physiologically relevant context for their innate immunomodulatory activity in the dental pulp and their capability for pulp repair [31].

The chemokines expressed by MSCs have been reported to regulate the migration and function of immune cells to maintain tissue homeostasis. BM-MSCs can rapidly express MCP1 in response to circulating TLR ligands or bacterial infection and induce monocyte trafficking into the bloodstream [95]. BM-MSC-derived CCL2 inhibits CD4^+^ T-cell activation by suppressing STAT3 phosphorylation and reversing symptomatic neuroinflammation in experimental autoimmune encephalomyelitis [96]. CCL2 and CXCL12 secreted by BM-MSCs acts as a heterodimer to upregulate IL-10 expression in CCR2^+^ macrophages in vitro, and CCL2 expression by MSCs is required for IL-10-mediated polarization of intestinal and peritoneal resident macrophages in vivo [97]. We also found that ionizing radiation could activate the cGAS-STING signaling pathway in MSCs and consequently lead to the upregulation of CCL5. CCL5 can then recruit macrophages to the lung to establish a microenvironment that is conducive to tumor metastasis [98].

#### EVs

MSCs also exert their therapeutic effects through the secretion of EVs. EVs are lipid-bound vesicles that are secreted into the extracellular space. The cargo of EVs consists of proteins, organelles and nucleic acids, which are responsible for the regulatory function of MSCs. The main subtypes of EVs include exosomes (50–100 nm in diameter), microvesicles (MVs; 0.1–1 μm in diameter), and apoptotic bodies (1 μm to 5 μm in diameter). MSCs have been shown to secrete at least 3 types of EVs, which can be differentially isolated based on their affinities for membrane lipid binding ligands and are likely to have different biogenesis pathways and different functions [99].

MSC-derived EVs have been shown to exhibit potent immunoregulatory properties. For example, human adipose MSC-derived exosomes inhibit the differentiation and activation of T cells and reduce T-cell proliferation and IFN-γ release [100]. The exosomes secreted by murine ADSCs can skew macrophages toward M2 polarization, which is mainly dependent on active STAT3 transferred by exosomes. Obese mice treated with ADSC-derived exosomes exhibited reduced adipose tissue inflammation, improved metabolic homeostasis, and resistance to obesity progression [101].

DSCs can also produce EVs to promote immune homeostasis. GMSCs even have a higher yield of small EVs than BM-MSCs and skin MSCs [96]. These small EVs are rich in IL-1RA. Mechanistically, GMSCs use the Fas/Fas-associated phosphatase-1/caveolin-1 complex to activate SNARE-mediated membrane fusion to secrete small EVs. Small EVs can accelerate wound healing in the gingiva [102]. Examination of inflammatory bone loss in a ligature-induced periodontitis mouse model showed that local injection of GMSC-derived exosomes significantly reduced periodontal bone resorption and the number of tartrate-resistant acid phosphatase-positive osteoclasts, and GMSCs treated with TNF-α exhibited further enhanced benefits [34]. Moreover, receptor activator of NF-κB ligand (RANKL) expression was upregulated by Wnt5a in periodontal ligament cells, and miR-1260b in exosomes could suppress the Wnt5a-mediated RANKL pathway and thus inhibit osteoclastogenesis. Cell-conditioned medium and purified EVs from PDLSCs reduced inflammatory damage in animal models of experimental autoimmune encephalomyelitis. The vesicles contained the anti-inflammatory cytokines IL-10 and TGF-β [103]. Exosomes secreted by PDLSCs were also shown to reduce bone loss in periodontitis [104].

MSCs undergo mitophagy in response to oxidative stress and promote the translocation of depolarized mitochondria to the plasma membrane *via* arrestin domain-containing protein 1-mediated microvesicles. The vesicles are then engulfed and reused by macrophages, resulting in enhanced macrophage bioenergetics and impaired TLR signaling [105]. MSC EV-mediated mitochondrial transfer also promotes an anti-inflammatory and highly phagocytic macrophage phenotype by enhancing macrophage oxidative phosphorylation [106].

Exosomal miR-182 delivery from BM-MSCs to macrophages converted inflammatory macrophages to the M2 phenotype by targeting TLR4, reducing inflammation levels in a mouse model of myocardial ischemia/reperfusion [107].

#### Apoptotic vesicles (apoVs)

During the treatment of immune diseases with MSCs, researchers found that despite being immunosuppressive, MSCs were undetectable after administration. Galleu. et al. found that MSCs were actively induced to undergo perforin-dependent apoptosis in a murine graft-versus-host disease model and that their apoptosis is essential to initiate MSC-induced immunosuppression. After infusion, recipient phagocytes engulf apoptotic MSCs and produce IDO, which mediates the immunosuppressive function of MSCs [108]. In addition, it was reported that calreticulin exposed on the surface of MSC-derived apoVs acted as a critical ‘eat-me’ signal mediating apoV efferocytosis by macrophages. MSC-derived apoVs can induce macrophage reprogramming at the transcriptional level, leading to the inhibition of macrophage accumulation and the transformation of macrophages toward an anti-inflammatory phenotype in T2D livers [109]. A recent study showed that MSC-derived apoVs could attenuate sepsis by switching neutrophil NETosis to apoptosis. ApoVs were recruited by bone marrow NETs *via* electrostatic charge interactions. FasL in apoVs could mediate the cell death pattern switch in neutrophils from NETosis to apoptosis and then inhibit the migration of neutrophils from bone marrow to distal organs, alleviating organ injury and sepsis [110].

#### T-cell death-mediated immunoregulation

It has been reported that BM-MSCs induce T-cell apoptosis *via* the FasL-dependent Fas pathway. Fas and FasL are members of the tumor necrosis factor (TNF)-receptor and TNF family, respectively. The binding of Fas to FasL results in the activation of a caspase cascade that initiates apoptosis. Apoptotic T cells subsequently trigger macrophages to produce high levels of TGF-β, which in turn leads to an increase in regulatory T cells and immune tolerance [111]. DPSCs can also induce activated T-cell apoptosis by the same mechanism and ameliorate inflammation-related tissue injuries when systemically infused into a murine colitis model [52]. Moreover, when cocultured with normal B cells, human PDLSCs suppressed B-cell proliferation, differentiation and migration. Mechanistically, human PDLSCs suppressed B-cell activation through cell-to-cell contact, which was mostly mediated by PD1 and PD-L1. Furthermore, the transplantation of allogenic human PDLSCs suppresses humoral immunity in a minipig periodontitis model [112].

### How MSCs/DSCs acquire and maintain their immunomodulatory properties

#### Cytokines and other secretory factors

**Fig. 3:**
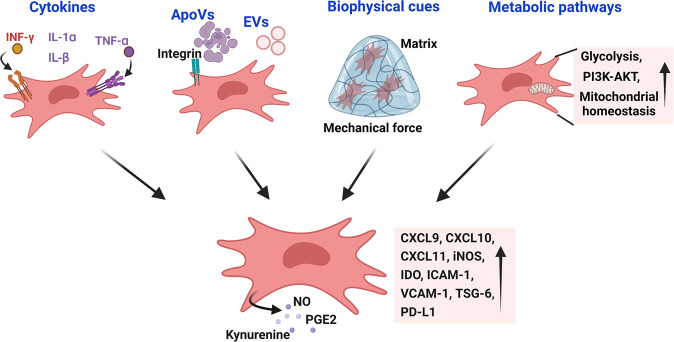
The mechanisms by which MSCs/DSCs acquire and maintain their immunomodulatory properties. The immunomodulatory properties of MSCs/DSCs are not constitutive but are induced by proinflammatory cytokines. Cytokines activate PI3K and AKT to initiate glycolysis, which is critically required for MSCs/DSCs to produce high levels of chemokines, adhesion molecules and effector molecules. Apoptotic bodies can endow MSCs/DSCs with enhanced immunomodulatory properties. The extracellular matrix and scaffold also support the immunomodulatory functions of MSCs/DSCs. MSCs/DSCs could also sacrifice themselves to fulfill their mission of immunosuppression

A landmark paper by Ren et al. demonstrated that the immunomodulatory properties of murine MSCs were not innate but were elicited by a set of inflammatory cytokines [69] (Fig. 3). T-cell chemotactic chemokines such as CXCL9, CXCL10, and CXCL11 can be upregulated over one million-fold. Nos2, which encodes iNOS, is upregulated to a similar extent and inhibits T cells. It should be noted that the immunoregulatory effects of MSCs are most efficiently elicited by a combination of a set of cytokines. Among these cytokines, IFN-γ appears to be indispensable for MSCs to acquire maximal immunosuppressive properties, although the concomitant presence of any of three other proinflammatory cytokines, TNF-α, IL-1α or IL-β, is also required [69]. Interestingly, instead of using NO as the effector to inhibit T cells by murine MSCs, human MSCs primarily use IDO to exert their inhibitory effect on T cells [113, 114]. MSCs also require the expression of adhesion molecules to exert their immunoregulatory effects [115]. The expression levels of ICAM-1 and VCAM-1 on MSCs were greatly upregulated in response to IFN-γ and inflammatory cytokines (TNF-α or IL-1). The immunosuppressive effect was significantly attenuated when the adhesion molecules were genetically deleted or functionally blocked, indicating the importance of cell‒cell contact in immunosuppression by MSCs. The inflammatory factor IL-17 was recently shown to synergize with IFN-γ and TNF-α to increase PD-L1 expression in MSCs [116]. Interestingly, the enhanced PD-L1 expression induced by IL-17 appeared to depend on iNOS because the upregulation of PD-L1 was not observed in deficient MSCs or in the presence of an iNOS inhibitor. It was recently reported that endothelial cell-derived IL-6 could activate IL-6R and STAT3 in DPSCs [117]. DPSCs in which STAT3 was knocked down were compromised in their vasculogenic potential when transplanted into immunodeficient mice.

While the aforementioned inflammatory cytokines are critical in inducing the immune modulatory properties of MSCs, the anti-inflammatory cytokine TGF-β has the opposite effect on the immunosuppressive effect of MSCs on anti-CD3-activated splenocytes. TGF-β can inhibit iNOS expression in a SMAD3-dependent manner. TGF-β produced by MSCs can act in an autocrine manner to reduce iNOS [118].

Single-cell transcriptome analysis of human oral mucosa in healthy individuals and patients with periodontitis was recently reported [119]. The transcriptome atlas revealed a complex cellular landscape of oral mucosal tissues consisting of epithelial, endothelial, immune and stromal cells. The study identified distinct epithelial and stromal cell populations with inflammatory signatures that promoted antimicrobial defenses and neutrophil recruitment. Stromal cells are particularly active in recruiting neutrophils in periodontitis. However, the exact stimuli that activate stromal cells to produce chemokines for neutrophils in periodontitis remain to be characterized.

#### Apoptotic vesicles and metabolites

In the human body, billions of cells undergo apoptosis every day, and a large number of apoptotic bodies need to be cleared to maintain tissue homeostasis [120]. Apoptotic cells release nucleotides as ‘find-me’ signals for clearance by phagocytes [121]. It was recently shown that apoptotic cells release metabolites, such as ATP, spermidine and creatine, in a regulated manner [122]. Uptake of these apoptotic bodies by phagocytes, which is also known as efferocytosis, has long been known to confer anti-inflammatory properties on recipient cells [123–125]. It appears that MSCs also need to feed on apoptotic bodies to maintain their stemness [126]. When the formation of apoptotic bodies was reduced, as in Fas-deficient MRL/*lpr* and Caspase 3^−/−^ mice, the self-renewal and osteogenic/adipogenic differentiation of MSCs was significantly impaired. The infusion of exogenous apoptotic bodies rescued these impairments and ameliorated the osteopenia phenotype in MRL/*lpr*, Caspase 3^−/−^ and ovariectomized mice. MSCs could engulf apoptotic bodies *via* integrin αvβ3 and reuse the apoptotic body-derived ubiquitin ligase RNF146 and miR-328-3p to inhibit Axin1 and thereby activate the Wnt/β-catenin pathway [126]. In addition to maintaining MSC stemness, the apoptotic cells engulfed by MSCs could also increase the immunosuppressive properties of the latter [127]. The apoptotic cells stimulated MSCs to express COX2 and consequently produced more PGE2, which inhibited T-cell responses.

As discussed previously, MSCs produce abundant levels of EVs to influence immune cells and other types of cells. It is likely that MSCs may also take up circulating vesicles and change their function and behaviors accordingly. The metabolites released by vesicles, as well as those produced from other routes, could have an impact on the immunomodulatory functions of MSCs. Future studies should provide more insights into the effects of vesicles on MSCs.

#### Biophysical cues

Mechanical force plays a critical role in determining the fate and characteristics of PDLSCs. Alveolar bone osteocytes produce sclerostin, a Win inhibitor, to negatively regulate Gli1+ PDLSC activity. Sclerostin expression is inhibited by physiological occlusal force [16]. Force-treated PDLSCs exhibited increased proliferation and reduced differentiation into osteocytes and adipocytes. These cells also highly expressed cytokines and RANKL to promote the generation of osteoclasts and bone remodeling [128]. Mechanical force was also shown to increase the level of exosomal proteins in PDLSCs to facilitate exosome internalization by macrophages and promote osteoclast differentiation [129]. These findings indicate that mechanical force may govern the immunomodulatory property of DSCs. Studies of the effect of the matrix and other biophysical properties on the function of other types of MSCs showed that biophysical cues could determine the immunoregulatory function of MSCs [130]. Many matrix biophysical parameters, such as fiber orientation, stiffness, dimensionality, and viscoelasticity, could impact the immunomodulatory function of MSCs.

A recent study showed that MSCs on a soft matrix produce higher levels of immunomodulatory factors than those on a stiff matrix [131]. The expression of TSG-6, the key mediator of the immunoregulatory effect of MSCs, was mechanosensitively regulated by the MAPK and Hippo signaling pathways and the downstream AP1 complex. Interestingly, when MSCs were exposed to various grades of wall shear stress within a scalable conditioning platform, their immunomodulatory potential was enhanced independent of classical proinflammatory cytokines, as evidenced by increased production of PGE2 and IDO1, as well as the suppression of TNF-α and IFN-γ production by activated immune cells [132]. Another study showed that compared with exosomes generated by MSCs in 2D culture, those produced by MSCs in 3D culture (3D-exos) exhibited enhanced anti-inflammatory effects in a ligature-induced model of periodontitis by restoring the Th17 and Treg balance in inflamed periodontal tissues [133]. 3D-exos were shown to be enriched in in miR-1246, which can suppress the expression of Nfat5, which promotes Th17 cell polarization. Local injection of 3D-exos attenuated experimental colitis. These findings indicate that biophysical conditions can have a great impact on the immunomodulatory properties of MSCs.

Anisotropic silk protein nanofiber-based hydrogels were developed to mimic the physical microenvironment inside the blastocele and were more effective in sustaining the stemness of mouse embryonic stem cells (mESCs) than the classical recipe containing leukemia inhibitory factor and mouse embryonic fibroblasts (MEFs) [134]. The mESCs on hydrogels could achieve pluripotency, developmental capacity, and germline transmission that was superior to those cultured with the standard protocol. Such biomaterials could stimulate the production of autocrine factors that maintain the pluripotency and propagation of ESCs. It is possible that the biophysical niches in which MSCs reside in situ play a critical role in determining their immunomodulatory functions. Future studies are expected to provide more insights into the role of biomechanical factors in the regulation of the immunomodulatory capacity of MSCs.

#### Metabolic pathways

The metabolic pathways required for MSCs to acquire and sustain their immunoregulatory functions have been studied recently. Inflammatory cytokines cause metabolic shifts in MSCs, including enhancing glycolysis and increasing fatty acid oxidation, but only interference with glycolysis but not fatty acid oxidation impairs IDO upregulation and the immunosuppressive function of MSCs [135, 136]. The ATP synthesis inhibitor oligomycin enhanced the immunosuppressive and therapeutic functions of MSCs [137]. It was shown that enhanced glucose turnover was associated with STAT1 glycosylation. Inhibiting the responsible O-acetylglucosamine (O-GlcNAc) transferase abolishes STAT1 activity and IDO upregulation [135]. It was recently shown that the PI3K-AKT signaling axis was rapidly activated and required for skewing toward glycolysis induced by TNF-α and IFN-γ. Moreover, MSCs expressing dominant-negative AKT were compromised in their therapeutic efficacy on IBD [136].

Maintaining proper mitochondrial turnover and function is critical for cellular homeostasis and many biological processes. It is well recognized that disrupted mitochondrial homeostasis contributes to neural degeneration. Likewise, mitochondrial dysfunction may compromise the functions of MSCs and lead to chronic inflammation-associated bone diseases, such as periodontitis and osteoarthritis. It was recently shown that chronic inflammation leads to excess Ca^2+^ transfer from the ER to mitochondria, and mitochondrial calcium overload further damages mitochondria. Due to the inhibition of mitophagy under chronic inflammatory conditions, damaged mitochondria continuously accumulate in MSCs [138]. Nanoparticles fabricated to capture Ca^2+^ around mitochondria reduced mitochondrial calcium flux, physiologically restoring the function of mitochondria and MSCs.

#### The cell death pathway of MSCs and immunomodulatory functions

While transplanted MSCs have been widely documented to exert potent immunomodulatory effects in various autoimmune diseases and graft-versus-host disease (GvHD), MSCs are rapidly cleared in recipients after their administration. The dynamics of transplanted MSCs in recipients indicate that MSCs do not need to persist very long to exert their therapeutic effects. It appears that their only mission is to deliver signals or messages to the recipients or to awaken host tissues. Using a murine model of GvHD, Dazzi and colleagues observed that MSCs actively underwent perforin-dependent apoptosis, which is essential for MSCs to have immunosuppressive functions [108]. In GvHD patients who received MSCs, only those with high cytotoxic activity against MSCs responded to the MSC infusion. The infusion of apoptotic MSCs generated ex vivo similarly exerted therapeutic effects. After the infusion, recipient phagocytes engulfed apoptotic MSCs and produced IDO1, the effector molecule for immunosuppression. Apoptosis was also shown to be required for DPSCs to have immunosuppressive functions in an allergic airway inflammation model [139]. A recent study showed that ferroptosis in MSCs induced by superparamagnetic iron oxide promoted efferocytosis by macrophages and thus enhanced the protective effect on septic mice, while these benefits were impaired after MSCs were treated with inhibitors of ferroptosis [140]. Whether and how the other fates of MSCs, such as pyroptosis and necroptosis, affect the immunosuppressive functions of MSCs remain to be explored.

### Perspectives on strategies for enhancing the therapeutic properties of MSCs/DSCs

With increased understanding of the mechanisms by which MSCs/DSCs exert their immunoregulatory effects and the extrinsic and intrinsic factors that stimulate and sustain the immunoregulatory functions of MSCs, it is possible to manipulate MSCs by various means to achieve enhanced therapeutic efficacies. We discuss some of the strategies that can increase MSC potency.

Like immune cells that need to be activated by intrinsic or extrinsic signals to achieve their mission, MSCs also need to be activated or induced to acquire their immunomodulatory function. Inflammatory cytokines produced by immune cells or other types of cells serve as essential stimuli to induce immunomodulatory functions [69]. Thus, MSCs and immune cells appear to form a negative feedback loop to keep immune responses in check. Interestingly, while TGF-β is generally regarded as an anti-inflammatory factor due to its ability to induce Treg differentiation and tissue fibrosis, it compromises the immunosuppressive function of MSCs. How to make the most use of this MSC-immune cell feedback loop to harness the immunomodulatory function of MSCs/DSCs clearly represents a promising venue for advancing MSC-based medicine.

Glycolysis is critical for the inflammatory function of various immune cells, and blocking glycolysis using 2-deoxy-D-glucose (2-DG) greatly compromises the proinflammatory response of immune cells. However, unlike in immune cells, this metabolic pathway serves to support the immunosuppressive function of MSCs. To behave as immunomodulators, MSCs need to be switched to a massive biosynthesis mode to produce a large number of chemokines and effector molecules in large quantities. Glucose understandably provides the carbon resources that are in great demand. Therefore, any means that can boost glycolysis in MSCs may increase the production of factors by MSCs. Because MSCs and inflammatory immune cells may compete for glucose in the tissue microenvironment, strategies that block glycolysis in immune cells but favor glucose uptake and glycolysis in MSCs will likely enhance their immunomodulatory functions. Metabolites derived from glycolysis, such as lactate, may also participate in the immunomodulatory function of MSCs. Mitochondria, where some crucial metabolic activities take place, may also contribute to the regulation of MSC immunomodulatory functions, and the exact roles may be elucidated in future studies.

Since the functions and fates of MSCs are determined by various biophysical cues they sense, it is possible to amplify the therapeutic functions of MSCs by implanting special MSC-laden scaffolds. MSCs seeded on certain matrices may possess functions that are not present in MSCs in monolayer cultures and last longer in vivo. An emerging field called developmental tissue engineering mimics morphogenetic processes during development to harvest biofunctional tissues and organs [141]. These approaches can be used to generate MSC-based materials that have long-lasting immunomodulatory functions in vivo.
